# COM7/386: ArztPartner AG: three steps strategy to a healthcare information network

**DOI:** 10.2196/jmir.1.suppl1.e20

**Published:** 1999-09-19

**Authors:** F Meise, N Hollweck

**Affiliations:** ^1^ArztPartner AG, MünchenGermany

**Keywords:** Internet platform, Networks, Quality, Search Engines, Healthcare Management

## Abstract

ArztPartner strives to create a network of the providers and receivers of medical care in Germany. This network will use the innovative technologies of cyberspace to increase the quality and speed of interaction between all participants of the healthcare system. Important components of this network will be virtual communities for patients, online discussion forums for physicians and possibilities of online interaction between the two groups. In the long run, this platform will be a basis for providing medical care on-line. The three-step-strategy:

1. Establishing a sophisticated physician search system for patients: Building on the current search routine of ArztPartner (www.arztsucher.de), ArztPartner will significantly upgrade its search options with more intuitive user-interfaces.
2. Bringing the ArztPartner physician- and clinics network on-line and upgrading the general physician database: Currently ArztPartner is putting the key information concerning the physicians and clinics in its quality survey network in the internet. At the same time we are providing all physicians and clinics in Germany with the opportunity to present their profile in our online network.
3. Building virtual communities for specific diagnoses, creating online discussion spaces for physicians and encouraging interactions between the two groups: ArztPartner will establish virtual communities for the most common diseases, thus helping consumers to get convenient access to specific information sources and exchange information with fellow patients.

At the same time, we will provide the medical providers in our network with possibilities to exchange information with each other and to access relevant content. In the long run, we hope to connect consumers, physicians and clinics, thus creating a platform for effective medical information exchange through the whole process of medical care. The presentation will show the current status of these efforts as well as the specific questions associated with this strategy.

